# Effect of Ondansetron on Maternal Hypotension During Spinal Anesthesia With Ropivacaine for Cesarean Sections: A Randomized, Double-Blind Trial

**DOI:** 10.7759/cureus.65073

**Published:** 2024-07-22

**Authors:** Stavroula Karachanidi, Anteia Paraskeva, Polyxeni Theodosopoulou, Georgia Micha, ‪Chryssoula Staikou

**Affiliations:** 1 Department of Anesthesiology and Pain Medicine, Aretaieion University Hospital, National and Kapodistrian University of Athens, Athens, GRC; 2 Department of Anesthesiology, University Hospital of Ioannina, Athens, GRC

**Keywords:** cesarean section (cs), spinal, isobaric ropivacaine, obstetric anesthesia, hypotension

## Abstract

Introduction: Ondansetron, a selective 5-hydroxytryptamine 3 (5-HT3) receptor antagonist, has been proven to be effective in the prevention of spinal-induced hypotension for elective cesarean section.

Methods: A total of 138 primigravida parturients scheduled for elective cesarean section were randomly assigned to three groups. Groups ONDA4 and ONDA8, respectively, received 4 and 8 mg of ondansetron in 100 mL normal saline, before spinal anesthesia with 1.7 mL ropivacaine 0.75% and 15 mcg of fentanyl, whereas the CONTROL group received an equal volume of normal saline. Noninvasive blood pressure and heart rate were recorded upon arrival, before and after spinal injection, and thereafter every minute for a time period of 10 minutes along with total doses of phenylephrine (mcg) or ephedrine (mg). Time required for the spinal anesthesia to achieve a sensory and motor block at the T4 level and Bromage 3 scale respectively, as well as to regress to the T7 level and a Bromage 1 scale were noted. Maternal nausea/vomiting or shivering, umbilical artery pH, and neonatal Apgar score at 1 and 5 min were also recorded.

Results: There were no differences between groups in systolic, diastolic blood pressure, heart rate (p=0.355, p=0.550, p=0.474 respectively), doses of phenylephrine or ephedrine, (p=0.920, p=0.142 respectively), time for the block to reach T4 (p=0.889) and Bromage scale 3 (p=0.269), or to regress to T7 (p=0.273) and Bromage scale 1 (p=0.392), the incidence of nausea/vomiting (p=0.898/p=0.365), umbilical artery pH (p=0.739), neonatal Apgar score at 1 and 5 min (p=0.936 and p=0.907 respectively).

Conclusion: Our results showed no significant effect of two different doses of ondansetron, in preventing maternal hypotension, following spinal anesthesia with ropivacaine for cesarean section.

## Introduction

Spinal anesthesia has become the technique of choice for cesarean section, due to its fast onset and significantly lower anesthetic risks for the mother and the fetus. However, the sudden decrease in afterload, following lumbar sympathetic blockade, may cause hypotension and therefore fetal compromise since uteroplacental flow lacks autoregulation. Furthermore, sudden blood pooling in conjunction with heart contraction against an empty ventricle can provoke paradoxical firing of cardiac receptors responsible for the Bezold-Jarisch reflex (bradycardia and hypotension) which may put an additional risk to the hypotensive parturient [1,2].

Ropivacaine, a long-acting amide local anesthetic, with a similar structure and pharmacodynamic profile to bupivacaine [3,4], has been used for both pain relief during labor and for anesthesia in cesarean sections [5]. Having been described as less potent than bupivacaine, ropivacaine has a slower onset of action, but a shorter duration of both motor and sensory block that allows for rapid patient recovery.

Serotonin, acting on the ligand-gated ion channel 5-hydroxytryptamine 3 (5-HT3) receptor, has been shown to elicit the Bezold-Jarisch reflex, and there is an increased expression of these receptors on vagal afferent nerves in the heart [6-8]. Furthermore, animal studies have shown that substantia gelatinosa has an abundance of 5-HT3 receptors, which play a role in nociceptive stimulus propagation. There is also evidence that 5-HT3 inhibitors have a negative effect on spinal sensory and motor block [9,10].

Vasopressors along with co-loading have been proposed as the ideal regimen to prevent and treat hypotension due to sympathetic blockade [11]. Ever since the first case report of post-spinal cardiac arrest which was successfully managed with atropine and ondansetron [12], the efficacy of serotonin antagonists in attenuating maternal hemodynamic responses due to spinal anesthesia has been investigated meticulously. More specifically ondansetron, which is a potent selective serotonin (5-HT3) antagonist with a high safety profile, when administered in doses up to 8 mg, had possible favorable effects in parturients undergoing spinal anesthesia with bupivacaine [13].

Previously a study by Paraskeva et al. [14] investigated the interaction between ondansetron and spinal ropivacaine in males undergoing transurethral surgery with regard to spinal block characteristics. Nevertheless, neither this study nor any study with spinal ropivacaine, according to our literature search, did focus on the hemodynamic parameters of this interaction.

In our department ropivacaine is widely used for spinal anesthesia in cesarean sections; therefore, we designed a protocol with the purpose of studying a possible dose-related antagonistic effect of ondansetron on hypotension after spinal anesthesia with ropivacaine in cesarean sections. By comparing two different doses of ondansetron to a placebo, we evaluated the impact of ondansetron on hemodynamic parameters, on the spinal blockade as well as on neonatal parameters.

## Materials and methods

Our study was approved by the Ethics Committee of Aretaieion University Hospital, Athens, and was registered at ClinicalTrials.gov under registration number NCT03931863. The study was performed in accordance with the ethical standards laid down in the 1964 Declaration of Helsinki and its later amendments. The Consolidated Standards of Reporting Trials (CONSORT) guidelines for reporting randomized control trials were followed.

One hundred and thirty-eight primigravida parturient women, classified as American Society of Anesthesiologists (ASA) II, at 38-41 weeks of gestation, who presented for elective cesarean section, were randomly allocated in three groups after signing an informed consent. Exclusion criteria were parturient’s refusal, contraindications to spinal anesthesia (coagulation disorders, inflammation at the puncture site, allergy to local anesthetics), ondansetron allergy, body mass index >35 Kg/m^2^, height <158 cm, or > 170 cm, hypertensive disorders of pregnancy, cardiovascular insufficiency, parturients receiving selective serotonin reuptake inhibitors (SSRI), serotonin and noradrenaline reuptake inhibitors (SNRI) or treatment for migraine, and placental abnormalities.

Randomization was based on a computer-generated random number sequence (randomiser.org). Group allocation was concealed using sealed envelopes. An independent nurse not participating in the study prepared the solutions according to group allocation. All solutions had the same volume (100 mL N/S) and appearance; thus, the investigators, patients, and personnel involved were blinded to the intervention. Ten minutes before spinal anesthesia, groups ONDA4 and ONDA8 received 4 mg and 8 mg of ondansetron, respectively, in a 100 mL of normal saline 0.9% solution intravenously within five minutes, whereas the CONTROL group received an equal volume of normal saline 0.9% solution.

In the operating room, standard monitoring, which consisted of an electrocardiogram (ECG), noninvasive blood pressure (NIBP), and pulse oximetry (SpO2), was applied and two peripheral intravenous catheters 18 G were introduced, as per institutional practice metoclopramide (10 mg) plus cimetidine (250 mg) were given intravenously.

Spinal anesthesia was performed in the left lateral position, using a 27-gauge pencil point spinal needle, at L3-L4 intervertebral space, with 1.7 mL of 0.75% ropivacaine and 15 mcg of fentanyl (total volume 2 mL). An infusion of 500 mL hydroxyethyl starch 6%, was initiated at a rate of 25 mL/min. Noninvasive arterial blood pressure, heart rate, and hemoglobin saturation were recorded upon arrival in the operating room, before and after subarachnoid injection, and every minute thereafter for a total of 10 minutes.

Parturients were placed supine, with a 15-degree left uterine displacement to prevent aorto-caval compression. Following spinal injection, assessment of the block started at 1 min intervals and the total time needed to achieve a sensory block at T4 dermatome and a motor block at grade 3 Bromage scale was recorded. Sensory block was defined by sensing a temperature difference on the application of a cold alcohol swab to the skin, whereas motor block was defined using the Bromage scale (Bromage scale 0 = ability to move hip, knee, ankle, and toes; 1 = ability to fully flex foot and knee, but not the hip; 2 = ability to fully flex foot, but not the knee and the hip; 3 = total motor block). At the end of the operation and after assessing at 15-minute intervals, the time needed for the sensory block to recede to T7 dermatome and for the motor block to regress to a grade 1 Bromage scale was recorded. Delivery time, neonatal Apgar score at the first and fifth minute post-delivery, and umbilical cord pH were recorded.

Hypotension, defined as a systolic blood pressure below 100 mmHg, was treated with intravenous incremental doses of 20 mcg phenylephrine, when the heart rate was above 100 beats per minute, or with 5 mg ephedrine when the heart rate was less than 100 beats per minute. Bradycardia, defined as a heart rate below 60 beats per minute, was treated with 0.6 mg of atropine intravenously. For postoperative analgesia, 1 gram of paracetamol and 75 mg of diclofenac were given at the end of the operation intravenously, along with 0.1 mg/kg of morphine subcutaneously, as per the hospital’s protocol. Transfer to the ward was allowed after regression of sensory and motor blockade to T7 dermatome and to Bromage scale grade 1 respectively.

The primary outcome was a comparison of systolic blood pressure between groups in the first 10 minutes after spinal anesthesia. Secondary outcomes included ephedrine and phenylephrine consumption, spinal block characteristics, the occurrence of nausea, vomiting, or shivering, and neonatal outcomes.

Statistics

According to power analysis, in order to find a medium effect size (Cohen’s effect size 0.25) [15] in systolic blood pressure, a total sample of 138 subjects would be necessary for a power of 0.80 and an alpha error of 0.05. To compensate for dropouts, we included 144 parturients in total.

Data was analyzed with Statistical Package for the Social Sciences (IBM SPSS Statistics for Windows, IBM Corp., Version 26.0, Armonk, NY). Normality was assessed with the Kolmogorov-Smirnov test. Demographics - body weight excluded - time for the sensory block to reach T4 regress to T7 dermatome, time for the motor block to reach grade 3 Bromage scale or to regress to grade 1 Bromage scale, total dose of phenylephrine and ephedrine and umbilical artery pH, were analyzed with Kruskal-Wallis test. Body weight was analyzed with a t-test. Systolic, diastolic blood pressure, and heart rate were analyzed with analysis of variance (ANOVA) repeated measures. Finally, nausea, vomiting, shivering, and hemoglobin saturation were analyzed with the chi-square test.

## Results

**Figure 1:**
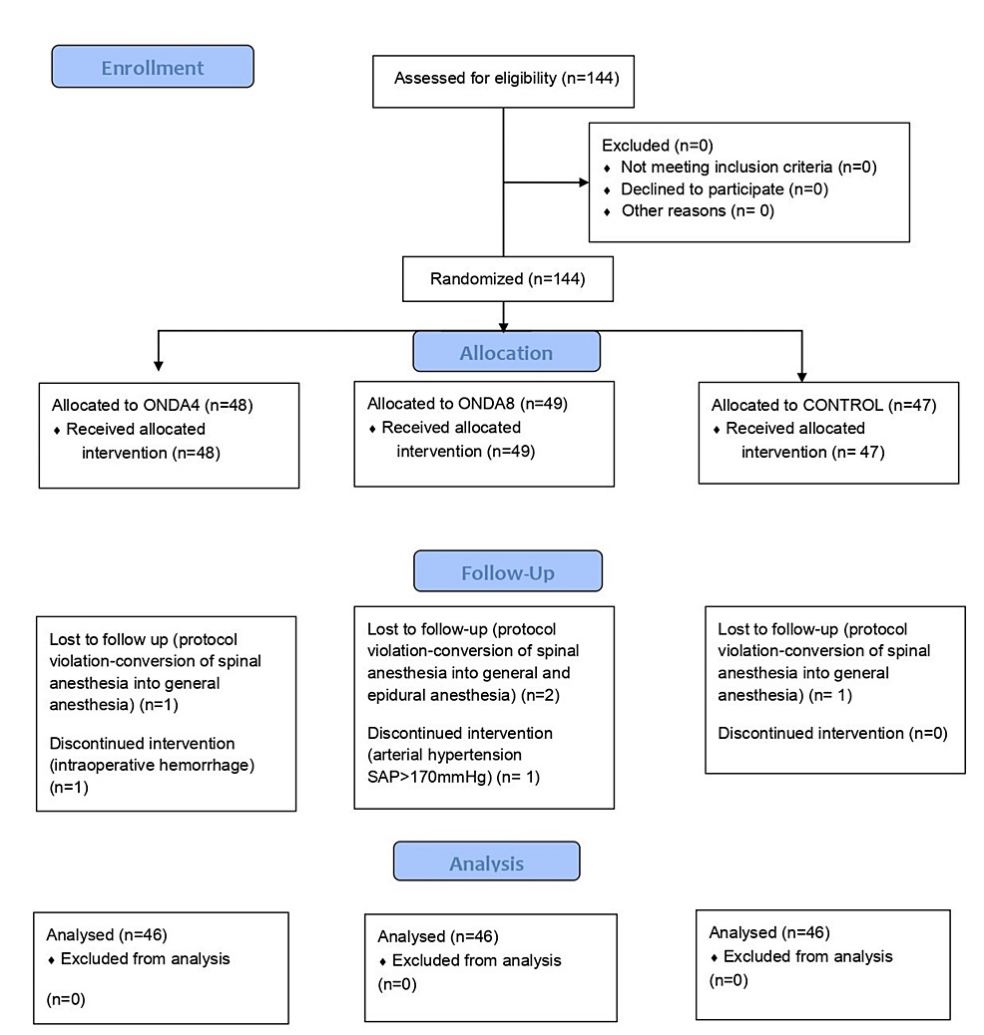
Flow diagram ONDA4: group ONDA4 received 4 mg ondansetron; ONDA8: group ONDA8 received 8 mg ondansetron; SAP: systolic arterial pressure

Data from 138 parturients were analyzed, as shown in the flow diagram (Figure 1). Demographic data, weeks of gestation, and time until delivery did not differ between the groups (Table 1).

**Table 1:** Demographic data of the patients Results are presented as mean (± standard deviation) with a significance level set at p < 0.05. ONDA4: group received 4 mg ondansetron; ONDA8: group received 8 mg ondansetron

Data	ONDA4 (n=46)	ONDA8 (n=46)	CONTROL (n=46)	P-value
Weight (kg)	81 (11)	78 (10)	77 (10)	0.210
Height (cm)	164 (4)	164 (4)	163 (4)	0.366
Age (years)	33 (5)	33 (5)	34 (4)	0.671
Gestational age (weeks)	38.7 (0.6)	38.6 (0.78)	38.9 (0.8)	0.063
Time to delivery (min)	12.5 (4.6)	12.5 (4.4)	13.9 (5.7)	0.575

**Figure 2:**
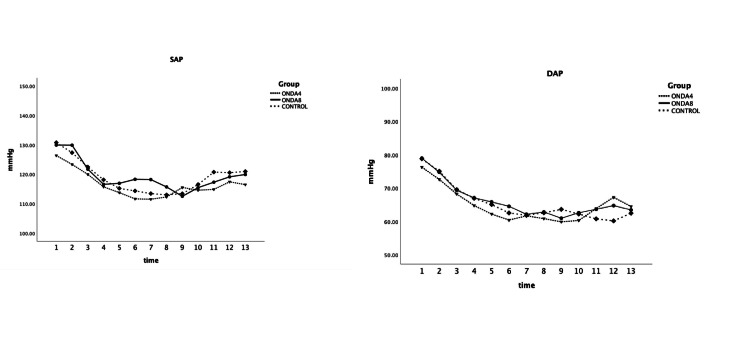
Systolic arterial pressure (SAP) and diastolic arterial pressure (DAP) were measured at selected time points: upon arrival in the operating room (1), one minute before spinal anesthesia (2), immediately after spinal injection (3), and at one-minute intervals up to 10 minutes (F=1.044 and P=0.355, F=0.6 and P=0.550) ONDA4: group received 4 mg ondansetron; ONDA8: group received 8 mg ondansetron

**Figure 3:**
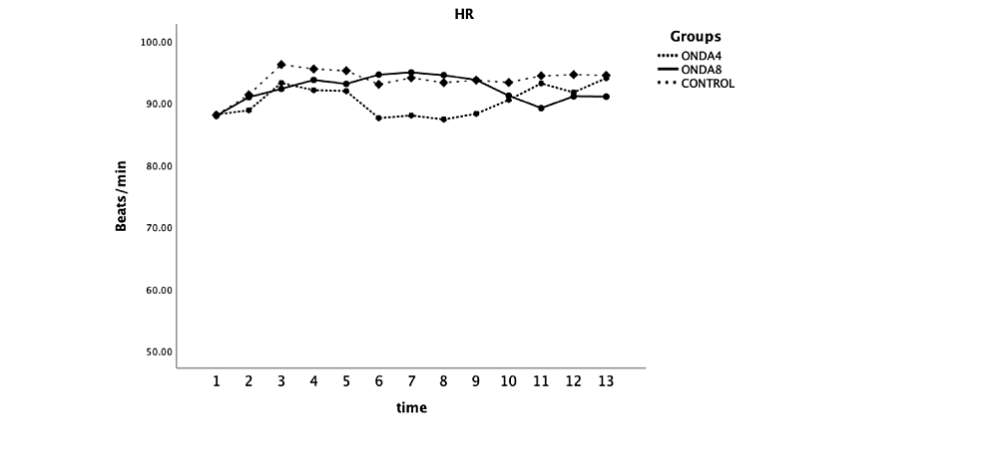
Heart rate (HR) was measured at selected time points: upon arrival in the operating room (1), one minute before spinal anesthesia (2), immediately after spinal injection (3), and at one-minute intervals up to 10 minutes (F=0.75 and P=0.474). ONDA4: group received 4 mg ondansetron; ONDA8: group received 8 mg ondansetron

No difference was found between groups in systolic and diastolic arterial blood pressure, or heart rate at any time point of recording (F=1.044 and P=0.355, F=0.6 and P=0.550, F=0.75, and P=0.474 respectively) (Figures 2-3).

Total doses of phenylephrine or ephedrine did not differ at any time during the study period (Table 2). Additionally, the number of patients requiring any type of vasopressor agent did not differ among the three groups (34 (73.9%) in ONDA4, 25 (54.3%) in ONDA8, and 32 (69.6%) in the CONTROL group, p=0.118). The three groups also did not differ in sensory or motor block characteristics (Table 3).

**Table 2:** Total phenylephrine in mcg and ephedrine in mg required perioperatively. Results are presented as mean (± standard deviation) with a significance level set at p < 0.05. ONDA4: group received 4 mg ondansetron; ONDA8: group received 8 mg ondansetron

	ONDA4	ONDA8	CONTROL	P-value
Phenylephrine (mcg)	17 (44)	15.6 (36)	16.1 (49.2)	0.920
Ephedrine (mg)	13.2 (13.1)	8.6 (11.3)	10.8 (13.5)	0.142

**Table 3:** Time (min) needed for the sensory block to reach the T4 level and to recede to the T7 level and for the kinetic block to reach the Bromage 3 and Bromage 1 scale Results are presented as mean (± standard deviation) with a significance level set at p < 0.05. ONDA4: group received 4 mg ondansetron; ONDA8: group received 8 mg ondansetron

	ONDA4	ONDA8	CONTROL	P-value
Time for sensory block to reach T4	4.36 (2.5)	4 (2)	4.31 (3)	0.889
Time for sensory block to recede to T7	104.3 (21)	117 (21.9)	11.5 (15.8)	0.273
Time for motor block to reach Bromage 3	3.3 (2)	3.2 (1.8)	3.7 (2)	0.269
Time for motor block to recede to Bromage 1	71 (17)	76 (21)	72 (18.3)	0.392

Three parturients had nausea in the ONDA4 and ONDA8 groups, while four were in the CONTROL group (P=0.898). One parturient experienced vomiting in the ONDA8 group (P=0.365). Seven parturients experienced shivering in the ONDA4 group and nine in the ONDA8 and the CONTROL group (P=0.819).

Neonatal umbilical pH was 7.33 (±0.34), 7.33 (±0.47), and 7.32 (±0.70) in group ONDA4, ONDA8, and CONTROL respectively (P=0.739). In both Apgar scores, at one minute (median 9 in each of the three groups) and at five minutes (median 10 in each of the three groups), no statistically significant difference was observed (P=0.936 and P=0.907 respectively). Parturients in all groups had a hemoglobin saturation at or above 98% during the study period.

## Discussion

Our study failed to reveal any effect of two different doses of ondansetron compared to placebo, on maternal hypotension after spinal anesthesia with ropivacaine in cesarean section.

Ropivacaine is being used efficiently for spinal anesthesia in cesarean section and in comparison, to bupivacaine, it seems to have less influence on the hemodynamics, allows for faster motor and sensory recovery, and provokes less adverse reactions (nausea, vomiting, and shivering) [3-5]. We chose to study the possible impact of ondansetron on the hemodynamic effects of spinal anesthesia with ropivacaine during cesarean, since there is a gap in literature regarding their interaction.

Our results are similar to the results of Ortiz-Gómez et al. who compared 2 mg, 4 mg, and 8 mg of ondansetron to a placebo and found no difference in the incidence of hypotension between parturients under spinal anesthesia with bupivacaine 0.5% plus fentanyl, or in phenylephrine and ephedrine requirements [16]. Similarly, no effect on hemodynamic parameters was observed by Oofuvong et al., when different doses of ondansetron were compared in cesarean section under spinal anesthesia with 10 mg hyperbaric bupivacaine and 20 mcg of fentanyl [17].

Karacaer et al. proved that 8 mg of ondansetron given five minutes before spinal anesthesia - with 10 mg of bupivacaine and 20 mcg of fentanyl - did not prevent the hypotension in parturients undergoing elective cesarean delivery, but the ondansetron group had consumed lower doses of norepinephrine [18].

A recent meta-analysis of 21 studies by Zhou et al. concluded that parturients under spinal anesthesia for cesarean section, who received ondansetron, experienced significantly lower incidence of bradycardia, nausea, and vomiting than the placebo group [19]. However, there was no difference in the incidence of hypotension, shivering, or pruritus between the groups. According to the authors, the significant heterogeneity of the studies (i.e. different techniques both spinal and epidural, different endpoints of the included studies with some of them focusing on nausea and vomiting as the primary endpoint), and the small number of randomized clinical trials included in some subgroup analyses were the limitations of their meta-analysis [19].

On the other hand, a meta-analysis of 10 studies conducted by Gao et al. confirmed the favorable effect of ondansetron in preventing hypotension due to spinal anesthesia, although among the included obstetric studies, heterogeneity was observed due to differences in defining spinal hypotension, the spinal anesthesia technique, the fluids and the vasopressors being used [20]. Heesen et al., in their meta-analysis of eight obstetric and nine non-obstetric studies, reported that ondansetron at doses ranging above 4 mg had a moderate effect in preventing hypotension and bradycardia, only in the obstetric population and again with a significant heterogeneity among the included studies [21]. Tubog et al., after conducting a meta-analysis of 13 obstetric and non-obstetric randomized trials, concluded that patients who received ondansetron had higher arterial blood pressure 15 to 20 min after spinal anesthesia [22].

More recently in a study conducted by Aksoy et al., it was documented that high doses of ondansetron (8 mg) or granisetron (3 mg) prevented hypotension in cesarean section under spinal anesthesia with 9 mg of isobaric bupivacaine [23]. Similar results were observed in a trial of a non-obstetric population, conducted by Mendonça et al., when 8 mg of ondansetron were given to patients undergoing spinal anesthesia achieved with 15 mg bupivacaine or more, along with adjuvant opioids [24]. In the study of Xiao et al., a significant reduction in the ED50, or the effective dose 50, of phenylephrine infusion was noted in parturients undergoing spinal anesthesia with 10 mg hyperbaric bupivacaine and sufentanil, when 4 mg of ondansetron were given 10 minutes before spinal anesthesia [25].

One reason for the lack of a significant difference in hemodynamic parameters between groups in our study might be associated with the slower onset of spinal block after ropivacaine in comparison to the more rapid onset achieved with bupivacaine [26]. This could result in a more gradual establishment of the sympathetic blockade and therefore the possible impact of ondansetron on hemodynamic parameters could be minimal. Another possible explanation is the fact that ropivacaine is correlated with a better hemodynamic profile than bupivacaine [26], thus the lower incidence of hemodynamic changes with ropivacaine, in general, may have not allowed for demonstrating a benefit with the use of ondansetron.

Many of the studies, that recorded a favorable effect of ondansetron on maternal hypotension due to spinal anesthesia, used a higher total intrathecal volume of bupivacaine (>2 mL) compared to the spinal volume of ropivacaine we used. Therefore, we might have observed a lower incidence of hypotension, which in turn might have obscured a potential beneficial effect of ondansetron on the hemodynamic consequences of the spinal block.

Regarding secondary endpoints such as characteristics of sensory or motor block, no difference was observed among the groups. This finding was consistent with the results of a previous study, where no difference was observed in the ondansetron group compared to placebo, regarding block characteristics in male patients presenting for transurethral surgery under spinal anesthesia with ropivacaine [14].

Moreover, no difference was observed in the occurrence of shivering and this finding is consistent with the results of the meta-analysis by Zhou et al. [19]. An absence of difference regarding nausea, vomiting, and neonatal outcomes was an expected finding, since during cesarean section under spinal anesthesia, all these endpoints are closely related to the incidence of hypotension, which did not differ among the groups.

To our knowledge, this is the first study that aims to investigate the interaction between a prophylactic administration of ondansetron and spinal anesthesia with ropivacaine, regarding hemodynamic changes during cesarean section. In previous studies, systematic reviews, and meta-analyses, bupivacaine was the local anesthetic used for spinal anesthesia in populations with high heterogeneity, while in our investigation spinal anesthesia was induced with ropivacaine.

The determination of spinal anesthetic dose was based on total height, since a positive correlation between total body height and vertebral length was documented in a study conducted by Lin et al. in Chinese parturients [27]. In an effort to limit variance within the study population, we confined parturient height in the range of 158-170 cm and used a standard dose of local anesthetic, in order to avoid not only an inefficient block, but also a high sensory blockade that could result in profound hypotension.

A limitation of our study is that instead of using the 20% reduction of the preoperative value or the mean arterial pressure below 65 mmHg to define hypotension, we used the cutoff point of 100 mmHg in systolic pressure based on previous literature [28-30]. Another limitation is that we used a noninvasive method for blood pressure measurements, whereas an arterial line would have provided us with beat-to-beat recordings. However, in our hospital, it is not a common practice to use an invasive technique for blood pressure measurements in non-high-risk cesarean sections.

## Conclusions

In conclusion, in our study’s setting, neither 4 mg nor 8 mg of intravenous ondansetron prevented hypotension in parturients undergoing spinal anesthesia with ropivacaine. Our study appears to be the first addressing the interaction between ropivacaine-induced neuraxial blockade and the preventive role of ondansetron for post-spinal hypotension. Further studies are needed in both pregnant and non-pregnant populations, with a greater emphasis on the pharmacokinetics of the administered drugs.
